# A prospective comparative analysis of the survival rates of conventional vs no-prep/minimally invasive veneers over a mean period of 9 years

**DOI:** 10.1007/s00784-021-04289-6

**Published:** 2021-12-20

**Authors:** Beata Smielak, Oskar Armata, Witold Bojar

**Affiliations:** 1 Department of Prosthodontics, Medical University of Lodz, ul. Pomorska 251, 92-213 Lodz, Poland; 2 Department of Conservative Dentistry and Endodontics, Medical University of Lodz, ul. Pomorska 251, 92-213 Lodz, Poland; 3 Private Practice, Witod Bojar Praktyka stomatologiczna, ul. Opaczewska 43/124b, 02-201 Warsaw, Poland

**Keywords:** No prep veneers, Minimally invasive veneers, Porcelain veneers

## Abstract

**Objectives:**

The present study compares the survival rates of 186 conventional and no-prep/minimally invasive porcelain veneers in 35 patients over a mean period of 9 years.

**Materials and methods:**

The veneers were placed on the incisors, canines, and premolars in 35 patients between January 2009 and December 2010. Fourteen patients received 84 conventional veneers, and 21 patients received 102 no-prep/minimally invasive veneers. The restorations were evaluated at baseline and after every 6 months until June 2019 based on modified United States Public Health Service criteria. The data was analyzed by using Wilcoxon–Breslow–Gehan and Taron–Ware tests. Kaplan–Meier survival and success curves were plotted for two groups of veneers. The results were compared by using the log rank test. A test probability of *P* < .05 was regarded as significant, while a test probability of *P* < .01 was considered to be statistically significant.

**Results:**

The mean survival rate, according to the Kaplan–Meier estimator, was 9.67% for conventional veneers and 100% for the no-prep or minimal prep veneers. A total of ten absolute failures were observed in six patients: eight restoration chipping/fractures, one debonding, and one fracturing of the tooth. Mean success rate time for conventional veneers without absolute or relative failures was 9.32 years, and 10.28 years for no-prep/minimally invasive veneers.

**Conclusions:**

Over a mean observation period of 9 years, the survival rate of no-prep/minimally invasive veneers exceed that of conventional veneers.

**Clinical relevance:**

No-prep/minimally invasive veneers appear very effective and should always be considered in certain clinical situations.

## Introduction

The advancement of dentistry technologies and the growing esthetic expectations of patients have led to increased interest in porcelain veneers. Such treatment has been greatly improved thanks to recent developments in the physical properties of ceramics and bonding systems [1]. Generally, no-prep/minimally invasive veneers tend to have a thickness of 0.2 to 0.5 mm [2–5], while conventional veneers range from 0.3 to 1.0 mm [6–12].

A recent trend in this field is the use of ultrathin, or *contact lens* veneers, which do not require invasive tooth preparation [13]. Despite being thin, they offer good color stability and have a permanent aesthetic effect [14]. In addition, the use of modern bonding systems enables good adhesion to porcelain, which allows for easy finishing and contouring in the gingival region [5]. While no-prep/minimally invasive veneers are biocompatible with dental substrates and are gentle to the periodontium, accumulate less bacterial plaque, and promote better oral hygiene. In addition, while conventional veneers require aggressive tooth preparation, this is not necessary for no-prep veneers, or only minor preparation within the enamel for minimally invasive veneers; however, additional orthodontic treatment may sometimes be required in cases of protrusion, a change in the position of the teeth, or severe crowding [14, 15].

The advantages of no-prep/minimally invasive veneers are that they are less painful or completely painless, there is often no need for anesthesia and impressions can be taken easily; in addition, there is no need for temporary restorations, which makes them readily accepted by patients who know that their natural teeth remain practically intact [5, 13–18].

The material of choice for the no-prep/minimally invasive veneers is feldspathic porcelain, which allows for the fabrication of very thin veneers from 0.2 to 0.3 mm; in comparison, thicker pressed ceramics have a thickness of 0.3 to 0.5 mm and require more aggressive reduction of dental structures [19, 20].

Many studies demonstrated the success of conventional veneers, but just few clinical studies are available for truly no-prep veneers [17, 21–27]. Moreover, comparative studies are extremely rare [1, 22–24, 28–32]. The aim of this paper is to provide a prospective comparative analysis of the survival rates of conventional and no-prep/minimally invasive porcelain veneers over a mean observation period of 9 years.

## Material and methods

A total of 35 consecutive patients requesting indirect ceramic veneers were recruited between January 2009 and December 2010. In total, 186 ceramic veneers were placed and evaluated. The final evaluation visits took place in June 2019. In 2009, only conventional veneers were being used; however, from January 2010, due to the development of new technologies and techniques, only no prep or minimally invasive veneers were used.

Before participating in the trial, all the patients were provided with informed consent forms approved by the ethics committee of the university institutional review board (ABR number: RNN/92/19/KE). Each patient was provided with information regarding alternative treatment options. The inclusion criteria were as follows: all subjects had to be at least 18 years old, able to understand and sign the informed consent form, physically and psychologically able to undergo conventional restorative procedures, with no active periodontal or pulpal diseases and with all cavities properly restored. The exclusion criteria were as follows: patients with no sufficient amounts of hard tissue, with deep class V or III restorations that may prevent the cementation with the use a rubber dam, and with strong discoloration were also disqualified.

The fillings were examined under an SOM 62 Ophthalmic Microscope (Karl Kaps GmbH & Co). In the case of secondary caries or leakage, the restorations were replaced. Patients with such parafunctions as bruxism (*n* = 2) were qualified for treatment after being informed of the risks associated with the procedure and that they would need to use a night-guard following treatment. Only teeth exhibiting defects comprising no more than one third of the incisal edge were treated with veneers. In addition, endodontically treated teeth were qualified for treatment following a positive X-ray examination (*n* = 10). The veneers were applied for the following indications: masking discolorations, such as discoloration as a result of endodontic treatment or use of tetracyclines, hypoplasia or hypercalcemia of enamel, and fluorosis, masking existing fillings of small class III, IV, and V cavities, closing small diastemas, reconstructing fractures and chipped-off enamel, changing the shape of teeth, minor displacements and rotations, revitalizing existing porcelain, and porcelain-metal restorations. Contraindications included major tooth fractures and severely damaged teeth.

After a thorough and accurate history including detailed information of the patient’s esthetic expectations, a series of photographs were taken of all the patients. Impressions were taken, diagnostic models were made, bite records were taken, future prosthetic restorations were simulated both by means of diagnostic wax-ups on models mounted in articulators, as well as with the help of DSD (Digital Smile Design) software. Each patient had a mock-up made from composite resin (Luxatemp; DMG) with the help of an index based on the wax-up. At this stage, the teeth were analyzed for their length with respect to the upper and lower lips, as well as for the correctness of their shape and proportions. Attention was also paid to function and phonetics. After checking the esthetics, the provisional restorations (mock-ups) were removed or left in place, depending on the preparation of the teeth.

The following conventional preparation depths were used: 0.1–0.2 mm cervically, 0.3–0.7 mm in the central part, 1–1.5 mm incisally, 0.5–0.7 mm palatally or lingually [8, 12, 14, 23]. In every case involving significant dentine exposure, a dentin bonding agent (DBA) was applied locally following acid etching and primer application [33, 34]. The DBA was applied to the freshly cut dentin following pre-treatment of the tooth and prior to taking impressions. In the case of no-prep or minimally invasive veneers, only minimal preparation of the enamel was needed, or none at all. The amount of enamel preparation was marked on the plaster cast. Minimal corrections were needed to reduce hard enamel tissue by between 0.2 and 0.3 mm. The patients were informed of the possible need to correct the position of their teeth by means of orthodontic treatment. The preparation depth was controlled using a silicone key made on the diagnostic wax-up.

Impressions were taken using a polyvinyl siloxane impression material (Express; 3 M ESPE GmbH). Prior to taking impressions for conventional veneers, a retraction cord was applied (Sil-Trax AS; Pascal Company, Inc.). This was not necessary in the case of no-prep veneers [20]. The placement of the retraction cord did not require an anesthetic in most cases. The veneers were made of feldspathic ceramic (Sakura Interaction; Elephant Dental B.V.) on refractory dies. All the veneers were prepared in the same dental laboratory.

After checking for marginal integrity, and confirming the correct shape and color, and receiving approval from both patient and dentist, the veneers were cemented into place. Prior to cementation, after cleaning with 99% isopropanol, they were etched with 5% hydrofluoric acid (IPS Ceramic etching gel; Ivoclar Vivadent) for 60 s and washed in alcohol in an ultrasonic bath for 5 min. After drying with oil-free compressed air, three layers of silane (Monobond S; Ivoclar Vivadent) were applied. Each layer was dried with hot air. After silanization, the bonding system (Panavia F 2.0; Kuraray) was applied. All the teeth to be veneered were isolated using the split rubber dam technique. Contour matrices (Contour-Strip, Ivoclar Vivadent) were placed interproximally with the help of wedges to create a smooth restoration outline in the cervical area.

The prepared teeth were first cleaned with fluoride-fee pumice (Pumice Flour; Kerr Dental) using a polishing brush (Coltene Whaledent; Altstatten); then, they were etched with 37% orthophosphoric acid (Etchant Gel; Ivoclar Vivadent) for 20 s. Once the acid had been rinsed out and the teeth dried with compressed air, the bonding system (Panavia F 2.0; Kuraray) was applied. Porcelain veneers were cemented using a dual-curing cement (Panavia F 2.0; Kuraray). The cement was placed by means of a flat plastic spatula and spread with a small brush. The cement was applied to the inner surfaces of the veneers. After full sitting, they were photopolymerized for only 3 s from the buccal aspect to ensure their stabilization. The excess cement was removed under a microscope with an explorer, metal strips, and dental floss.

The final polymerization was performed through glycerin gel (Oxyguard; Kuraray) with a curing lamp (Elipar DeepCure-L LED Curing Light, 3 M ESPE). Each side was light cured for 20 s. Following dual curing, an additional 5 min were allowed for self-curing, following which the gel was rinsed out. The excess cement was removed under a microscope. Then the bite was checked for correct contacts in centric and eccentric occlusion. After the adjustments, photographs were taken. A follow-up visit was scheduled within the following 24 h. The restorations were evaluated at baseline and thereafter every 6 months by two calibrated observers who were blinded to the aim of this study in accordance with the modified United States Public Health Service (USPHS) criteria (Table 1) [35, 36]. Absolute failures were considered as those where chipping and fracture was such that they could not be repaired. Besides absolute failures, relative failures were also reported as small defects that did not affect the possibility to maintain the restoration in situ, for example, a restoration crack, minimal ceramic fracture, slight chipping of restorations that could be simply burnished in situ, and slight marginal discolorations. Patients were also obliged to report possible postoperative symptoms. The restorations were inspected using a dental mirror and probe and evaluated to achieve a final score. Digital photographs were taken after placement of the veneers and during follow-up visits.

**Table 1:** List of modified United States Public Health Service criteria used for the clinical evaluations of the restorations

Category	Score	Criteria
Marginal Adaptation	0	Smooth margin
	1	All margins closed or possess minor voids or defects (enamel exposed)
	2	Obvious crevice at margin, dentin or base exposed
	3	Debonded on one side
	4	Debonded on both sides
Color match	0	Very good color match
	1	Good color match
	2	Slight mismatch in color or shade
	3	Obvious mismatch, outside the normal range
	4	Gross mismatch
Marginal discoloration	0	No discoloration evident
	1	Slight staining, can be polished away
	2	Obvious staining, cannot be polished away
	3	Gross staining
Surface roughness	0	Smooth surface
	1	Slightly rough or pitted
	2	Rough, cannot be refinished
	3	Surface deeply pitted, irregular grooves
Fracture of restoration	0	No fracture
	1	Minor crack lines over restoration
	2	Minor chippings of restoration (1/4 of restoration)
	3	Moderate chippings of restoration (1/2 of restoration)
	4	Severe chippings (3/4 restoration)
	5	Debonding of restoration
Fracture of tooth	0	No fracture of tooth
	1	Minor crack lines in tooth
	2	Minor chippings of tooth (1/4 of crown)
	3	Moderate chippings of tooth (1/2 of crown)
	4	Crown fracture near cemento-enamel junction
	5	Crown-root fracture (extraction)
Wear of restoration	0	No wear
	1	Wear
Wear of antagonist	0	No wear
	1	Wear
Wear of antagonist	0	No wear
Caries	0	No evidence of caries along the margin of the restoration
	1	Caries evident continuous with the margin of the restoration
Postoperative sensitivity	0	No symptoms
	1	Slight sensitivity
	2	Moderate sensitivity
	3	Severe pain

Statistical analyses were performed based on PQStat statistical software, version 1.6.4.110. The data was analyzed by using Wilcoxon–Breslow–Gehan test chi^2^ = 12.8494, *df* = 1, *p* = 0.0003, and Taron–Ware test chi^2^ = 12.8853, *df* = 1, *p* = 0.0003. Kaplan–Meier survival and success curves were plotted for two groups of veneers. The two sets of results were compared using the log rank test, and the power of this test was calculated A test probability of *P* < 0.05 was regarded as significant, while a test probability of *P* < 0.01 was considered to be statistically significant.

## Results

**Fig. 1:**
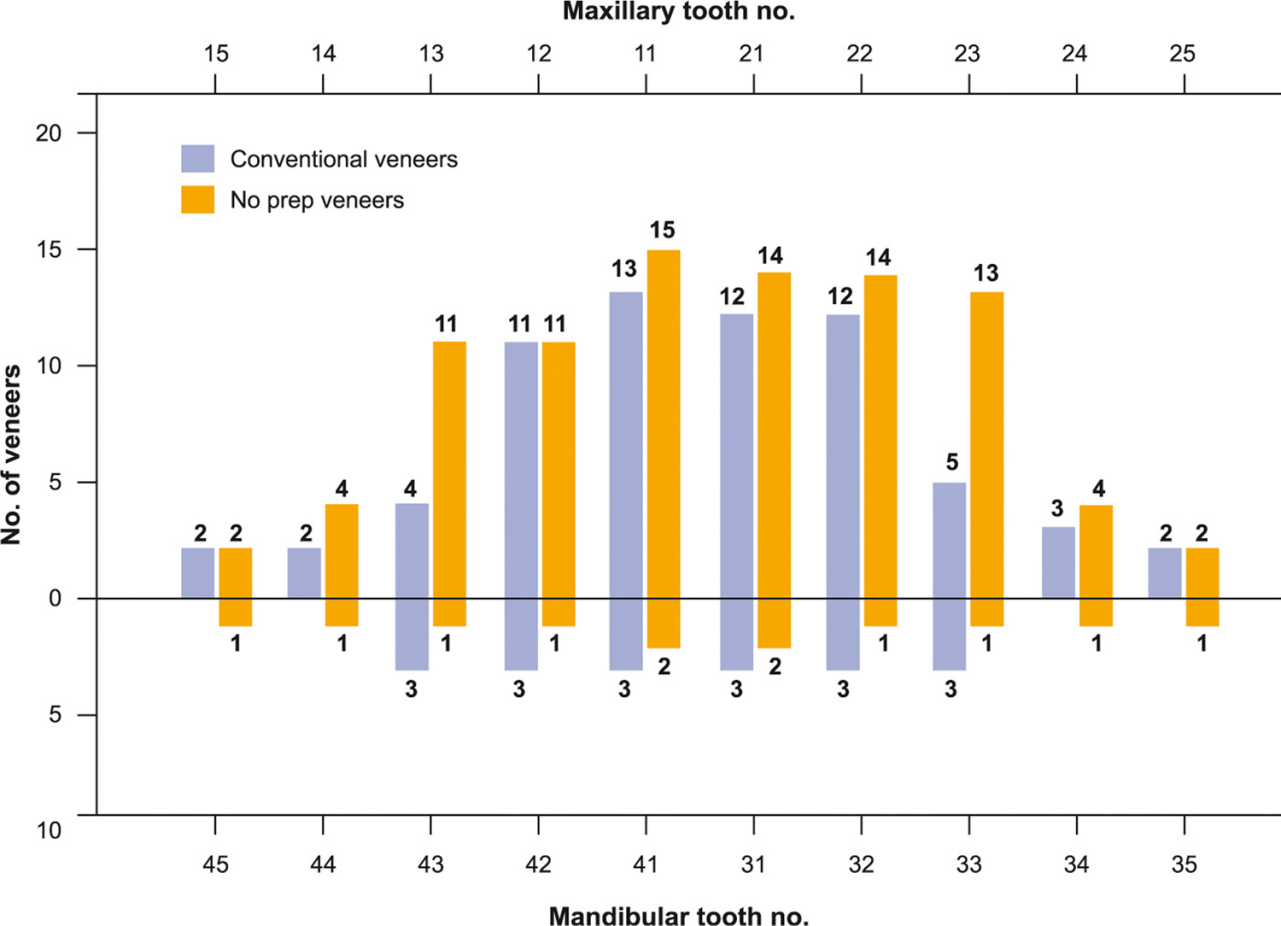
Numbers of conventional veneers and no-prep veneers (*n* = 186) placed on specific teeth. Veneers placed on maxillary teeth (*n* = 156) are represented by bars above the bisecting line; veneers placed on mandibular teeth (*n* = 30) are represented by bars below the bisecting line

In total, twenty recalls were performed after baseline measurements, and no drop-out was experienced, yielding to the evaluation of 186 veneers. A total of 35 consecutive patients requesting indirect ceramic veneers (28 women, 7 men; the mean age was 45 years (range: 26–64 years)) were recruited. Eighty-four veneers were placed and evaluated after conventional tooth preparation, while 102 were cemented with a no-prep (*n* = 32) approach or after minimal preparation (*n* = 70). They were distributed on the maxilla as follows: 54 on the central incisors, 48 on the lateral incisors, 33 on the canines, 13 on the first premolars, and eight on the second premolars. In the mandible, they were distributed as follows: 10 on the central incisors, eight on the lateral incisors, eight on the canines, two on the first premolars, and two on the second premolars (Fig. 1).

**Fig. 2:**
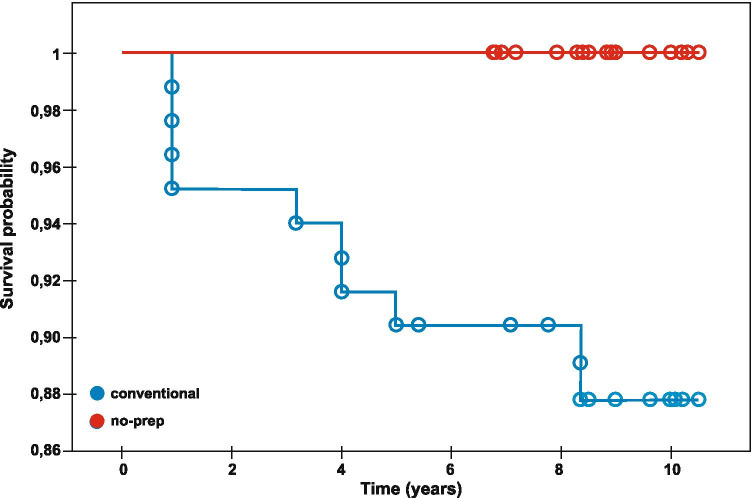
Kaplan–Meier survival curve of the cumulative survival of veneers depending on type of veneers

The mean time of observation was 9.06 years; shortest being 6.75 years, and the longest being 10.5 years, respectively. The mean survival rate, according to the Kaplan–Meier estimator, was 9.67% for conventional veneers and 100% for the no-prep or minimal prep veneers (Table 2, Fig. 2). A total of 10 absolute failures was observed: chipping/fracturing of restorations (*n* = 8), debonding (*n* = 1), and fracturing of the tooth (*n* = 1).

**Table 2:** Kaplan–Meier cumulative survival table for conventional veneers in situ for up to 11 years

Interval	Censored	Failure events	Entered	At risk	Failure events proportion	Censored proportion	Cumulative survival proportion	Probability density	Hazard rate	Standard error of cumulative survival	Standard error of probability density	Standard error of hazard rate
[0;1)	0	4	83	83	0.0482	0.9518	1	0.0482	0.0494	0	0.0235	0.0247
[1;2)	0	0	79	79	0	1	0.9518	0	0	0.0235	0	0
[2;3)	0	0	79	79	0	1	0.9518	0	0	0.0235	0	0
[3;4)	0	1	79	79	0.0127	0.9873	0.9518	0.012	0.0127	0.0235	0.012	0.0127
[4;5)	0	2	78	78	0.0256	0.9744	0.9398	0.0241	0.026	0.0261	0.0168	0.0184
[5;6)	3	1	76	74.5	0.0134	0.9866	0.9157	0.0123	0.0135	0.0305	0.0122	0.0135
[6;7)	0	0	72	72	0	1	0.9034	0	0	0.0325	0	0
[7;8)	3	0	72	70.5	0	1	0.9034	0	0	0.0325	0	0
[8;9)	12	2	69	63	0.0317	0.9683	0.9034	0.0287	0.0323	0.0325	0.02	0.0228
[9;10)	42	0	55	34	0	1	0.8747	0	0	0.0372	0	0
[10;11]	13	0	13	6.5	0	1	0.8747			0.0372

**Fig. 3:**
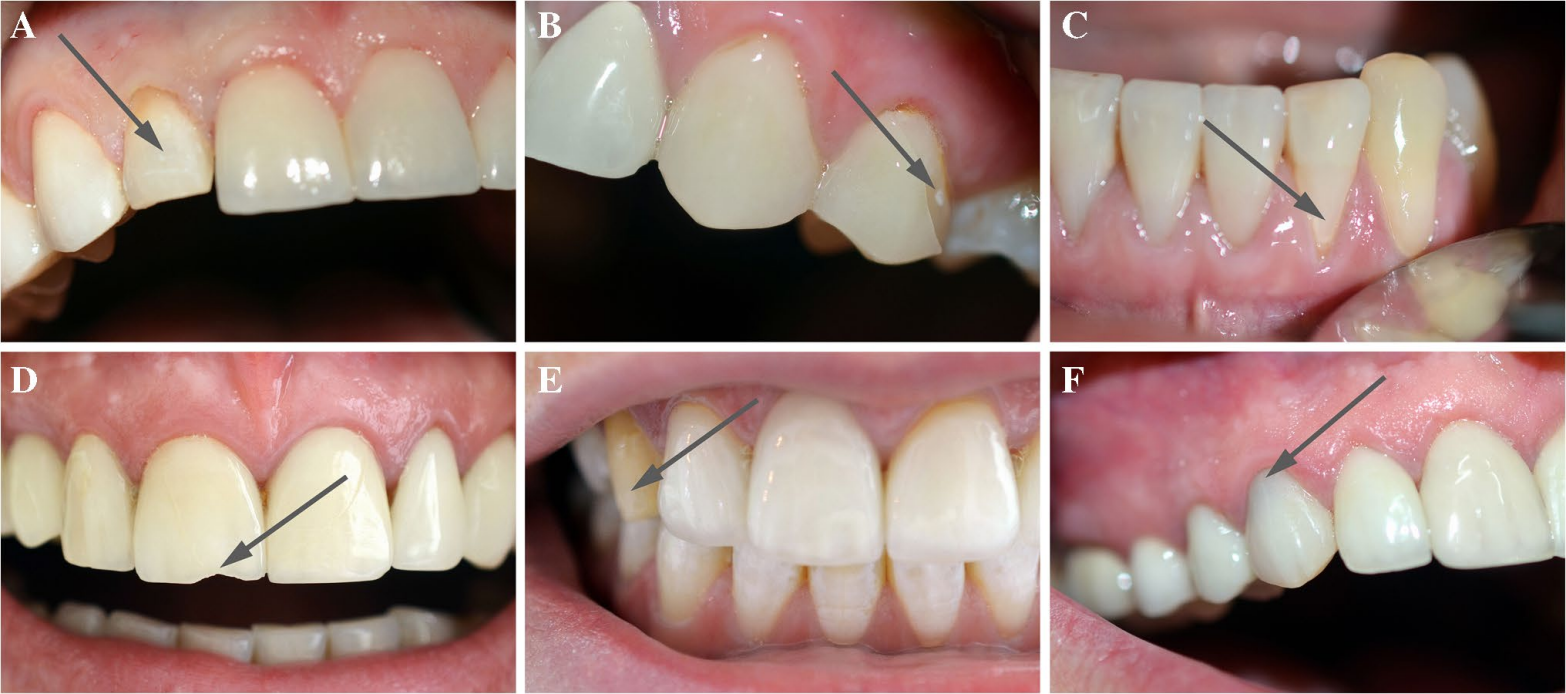
**A** Fracture of the laminate veneer on tooth 12. **B** Fracture of the laminate veneer on tooth 24. **C** Fracture of the laminate veneer on tooth 32 at the cervical area. **D** Cohesive chipping of the laminate veneer at the incisal edge of the ceramic on tooth 11 and multiple crack lines caused by trauma visible on veneers bonded to teeth 12, 21, and 22. **E** Adhesively debonded veneer from tooth 13. **F** Visible marginal discoloration on tooth 13

In addition, four patients demonstrated absolute failures, most of which were noted 18 months after placement: these were associated with a chipped incisal edge in veneers on teeth 12 (Fig. 3A) and 22, distal surface veneer on tooth 24 (Fig. 3B), and in the cervical area veneer on tooth 32 (Fig. 3C). The chipping occurred as a cohesive failure in the ceramic. One patient reported biting on a cherry seed, another did not use the night-guard given after treatment, and a third did not give a reason; in the last case, the patient had demonstrated a large composite filling in a class V cavity in tooth 32.

Following this, in two of the patients, fractures were observed after 48 and 100 months, respectively. One patient reported a fractured two veneers on central upper incisors after biting on a bite stabilizer while being positioned for the panoramic X-ray. The other broke four veneers, viz. 12–22, after hitting the anterior teeth with a ceramic cup (Fig. 3D). Two of the veneers was replaced (11 and 21), while the others (21, 22) were repaired chair-side with composite material. Another patient fractured tooth no. 12 after biting on a plum seed about 36 months after cementation. The veneer was replaced with a zirconia crown. One veneer debonded due to an adhesive failure between the tooth and the luting cement 60 months after placement (Fig. 3E). The veneer was bonded onto tooth 13 with no existing restorations. As the patient had lost the old laminate veneer, it was replaced with a new one of the same kind.

**Fig. 4:**
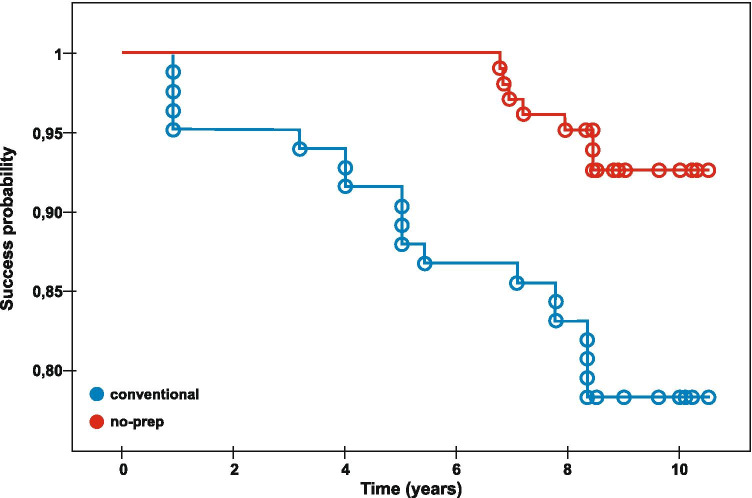
Kaplan-Meier success curve of the cumulative success of veneers depending on the presence of absolute or relative failures

Apart from absolute failures, relative failures were observed. Relative failures did not affect survival rate but decreased success rate. Mean success rate time for conventional veneers without absolute or relative failures was 9.32 years, and 10.28 years for no-prep/minimally invasive veneers (Tables 3 and 4). The Kaplan–Meier survival curve of the cumulative survival of veneers depending on the type of veneers and the presence of absolute or relative failures is presented in Fig. 4. An overview of failure characteristics is presented in Table 5.

**Table 3:** Kaplan–Meier cumulative success rate table for conventional veneers with absolute or relative failures for up to 11 years

Interval	Censored	Failure events	Entered	At risk	Failure events proportion	Censored proportion	Cumulative survival proportion	Probability density	Hazard rate	Standard error of cumulative survival	Standard error of probability density	Standard error of hazard rate
[0;1)	0	4	83	83	0.0482	0.9518	1	0.0482	0.0494	0	0.0235	0.0247
[1;2)	0	0	79	79	0	1	0.9518	0	0	0.0235	0	0
[2;3)	0	0	79	79	0	1	0.9518	0	0	0.0235	0	0
[3;4)	0	1	79	79	0.0127	0.9873	0.9518	0.012	0.0127	0.0235	0.012	0.0127
[4;5)	0	2	78	78	0.0256	0.9744	0.9398	0.0241	0.026	0.0261	0.0168	0.0184
[5;6)	0	4	76	76	0.0526	0.9474	0.9157	0.0482	0.0541	0.0305	0.0235	0.027
[6;7)	0	0	72	72	0	1	0.8675	0	0	0.0372	0	0
[7;8)	0	3	72	72	0.0417	0.9583	0.8675	0.0361	0.0426	0.0372	0.0205	0.0246
[8;9)	10	4	69	64	0.0625	0.9375	0.8313	0.052	0.0645	0.0411	0.0253	0.0322
[9;10)	42	0	55	34	0	1	0.7794	0	0	0.046	0	0
[10;11]	13	0	13	6.5	0	1	0.7794			0.046

**Table 4:** Kaplan–Meier cumulative success table for no-prep /minimally invasive veneers with absolute or relative failures for up to 11 years

Interval	Censored	Failure events	Entered	At risk	Failure events proportion	Censored proportion	Cumulative success proportion	Probability density	Hazard rate	Standard error of cumulative survival	Standard error of probability density	Standard error of hazard rate
[0;1)	0	0	103	103	0	1	1	0	0	0	0	0
[1;2)	0	0	103	103	0	1	1	0	0	0	0	0
[2;3)	0	0	103	103	0	1	1	0	0	0	0	0
[3;4)	0	0	103	103	0	1	1	0	0	0	0	0
[4;5)	0	0	103	103	0	1	1	0	0	0	0	0
[5;6)	0	0	103	103	0	1	1	0	0	0	0	0
[6;7)	0	3	103	103	0.0291	0.9709	1	0.0291	0.0296	0	0.0166	0.0171
[7;8)	0	2	100	100	0.02	0.98	0.9709	0.0194	0.0202	0.0166	0.0136	0.0143
[8;9)	50	2	98	73	0.0274	0.9726	0.9515	0.0261	0.0278	0.0212	0.0182	0.0196
[9;10)	19	0	46	36.5	0	1	0.9254	0	0	0.0275	0	0
[10;11]	27	0	27	13.5	0	1	0.9254			0.0275

**Table 5:** Overview of failure characteristics

	Conventional			No-prep/minimally preparation		
	*N*	% Total veneers	% Absolute or relative failures	*N*	% Total veneers	% Absolute or relative failures
Crack in the ceramic	1	1.2%	5.56%	2	1.94%	28.57%
Caries	0	0%	0%	1	0.97%	14.29%
Chipping	3	3.61%	16.67%	2	1.94%	28.57%
Marginal discoloration	1	1.2%	5.56%	0	0%	0%
Debonding	5	6.02%	27.78%	0	0%	0%
Fracture of the ceramic	7	8.43%	38.89%	2	1.94%	28.57%
Fracture of tooth	1	1.2%	5.56%	0	0%	0%

According to USPHS criteria, slight fractures of restorations were noted on three of the 74 conventional veneers and on two of the no-prep/minimally invasive veneers (USPHS criteria, Fractures of restoration-Scores 2) 85 to 101 months after cementation (Table 6). The complications were observed in the upper incisors. Another slight complication was the veneer debonding from the upper canines, which was observed for conventional veneers after between 60 and 93 months (*n* = 4). It is likely that restoration overloading and debonding were caused by the activity of strong lateral extrusion contacts on the veneers due to canine guidance. After cleaning the cementation surfaces, the debonded veneers were recemented using the same adhesive protocols. In one case, slight marginal discoloration was observed after cementation (USPHS criteria, Marginal Discoloration-Score 2) and the conventional veneer was deboned from tooth 13 (Fig. 3F). The adhesive protocol used at the emergency visit was unknown because the patient. In another case, 72 months after cementation conventional veneer caries on the distal surface of tooth 23 was observed (USPHS criteria, Caries-Score 1). After preparation, the tooth was repaired chair-side with composite material.

**Table 6:** Summary of USPHS evaluations at baseline and at the final recall

Criteria	Baseline			Final recall	
	Conventional (*n* = 84)		No prep (*n* = 102)	Conventional (*n* = 74)	No prep (*n* = 102)
Marginal adaptation	0	84	102	74	102
	1	-	-	-	-
	2	-	-	-	-
	3	-	-	-	-
	4	-	-	-	-
Color match	0	84	102	74	102
	1	-	-	-	-
	2	-	-	-	-
	3	-	-	-	-
	4	-	-	-	-
Marginal discoloration	0	84	102	73	102
	1	-	-	-	-
	2	-	-	1	-
	3	-	-	-	-
Surface roughness	0	84	102	74	102
	1	-	-	-	-
	2	-	-	1	-
	3	-	-	-	-
Fracture of restoration	0	84	102	71	102
	1	-	-	-	-
	2	-	-	-	-
	3	-	-	3	2
	4	-	-	-	-
	5	-	-	-	-
Fracture of tooth	0	84	102	71	102
	1	-	-	-	-
	2	-	-	-	-
	3	-	-	3	2
	4	-	-	-	-
	5	-	-	-	-
Wear of restoration	0	84	102	74	102
	1	-	-	-	-
Wear of antagonist	0	84	102	74	102
	1	-	-	-	-
Caries	0	84	102	74	102
	1	-	-	-	-
Postoperative sensitivity	0	84	102	74	102
	1	-	-	-	-
	2	-	-	-	-
	3	-	-	-	-

## Discussion

A systematic review by Morimoto [37] found an estimated overall cumulative survival rate for conventional feldspathic porcelain veneers of 87%. The median of the maximum follow-up times for the studies was eight years (range: 1.7 to 20 years). These findings were in agreement with Kreulen et al. [30] and Layton et al. [28], who reported a survival rate ranging from between over 90% and 95% over a period of between 3 and 10 years. In the present study, a low rate of complications was found for the following situations: debonding (2%), fracture/chipping (4%), caries (1%), severe marginal discoloration (2%), and endodontic problems (2%).

Feldspathic no-prep veneers have generally offered satisfactory performance, expressed in the form of a success rate range between 85 and 95% [17, 19, 37, 38]. In one study with an observation period of 2.5 years, 11 failures were observed out of 180 veneers: a 95% success rate [36]. However, the veneers in the study were thicker than the ultrathin veneers used in the present study: they had a mean thickness of 1 mm compared to 0.2–0. mm. The study also recommended no preparation of incisal edges if possible, assuming the result is aesthetically acceptable; has was a key principle in the present study. However, unlike the present study, an increased risk of failure was observed when veneers were placed on non-vital teeth.

Strassler and Ibsen [32] investigated 30 patients with 167 no-prep veneers. After 20 years, a clinical success rate of 94% was reported. Failures were mainly associated with chipping and cracking in stress-bearing areas. Elsewhere, a success rate of 91% was observed in a 10-year study of 191 ultrathin veneers measuring 0.3–0.5 mm [24], and a similar success rate was noted by Nordbø [8] after 3 years of observation of 135 laminate veneers in 41 patients. A slightly lower success rate of 85% was observed after 7 years in 50 patients in another study; however, this may be due to the fact that the veneers were thicker, for example just under 1 mm [30]. The latter findings are comparable to those of the present study, where the success rate of the thicker conventional veneers is 88%. In contrast, a 100% success rate was achieved for no-prep/ minimally invasive veneers, which appear to bond better with tooth enamel than with non-homogenous dentin.

The complications that affected conventional veneers included ceramic chipping, fracturing of crowns, and debonding. It is worth noting that one patient suffered four broken veneers as a result of trauma, and another two veneers were lost when an X-ray image was being taken. The same effect would have occurred in teeth not covered by veneers. On the other hand, especially in the case of trauma-related failures, failure cannot be solely attributed to poorer adhesion to dentin. A veneer bonded to a dentin substrate with higher elasticity may be exposed to higher stresses during loading, which could lead to an increased risk of fractures compared to veneers bonded to rigid enamel [21, 24, 30].

The tooth/porcelain complex may also display different compressive and flexural strength. Dentin has a much lower modulus of elasticity than porcelain, and hence, deeper preparation into it provides a less rigid base for restoration than enamel. Bonding to dentin results in much higher fracture rates than for enamel-supported restorations. Therefore, the reduction in dentin thickness observed after preparation may influence the life expectancy of the restoration [21, 39]. Enamel forms stronger mechanical bonds than dentin, which is less homogenous, contains humidity, and may possess sclerotic areas. In addition, if the remaining enamel is of higher quantity, the tooth will be stronger, since flexion of the tooth may be related to fractures and debonding [40, 41]. It is therefore much easier to get a good bonding interface between porcelain and enamel than between porcelain and dentin. Bonding to enamel results in less microleakage, caries, debonding, fractures, and discoloration. Secondary caries and marginal discoloration are less common with no-prep/minimally invasive veneers because all the margins are in positions that are relatively easy to clean [5, 14, 42–44]. More conservative preparation methods help preserve tooth vitality and reduce postoperative sensitivity [41].

In the present study, minor marginal defects and slight discolorations were not considered as absolute failures since they could easily be corrected. The patients did not notice them, and no intervention was required at the final evaluation visit. It is important to note that the complications associated with the use of conventional veneers were associated for the most part not with the chosen treatment method but rather in random events experienced by the patients. Such damage commonly occurs to teeth not covered with veneers as a result of traffic accidents, acts of violence, sport or biting on hard objects [29, 38]. Predisposing factors to failure in adults include endodontically-treated teeth, changes in dentine elasticity in patients over 40 years of age and the presence of numerous and extensive restorations [3, 17]. Particularly at risk are patients with parafunctions, such as bruxism, who do not follow the recommendations and do not wear a night-guard.

## Conclusions

Over a mean observation period of 9 years, the survival rate of no-prep/minimally invasive veneers exceed that of conventional veneers.
